# Influence of Brewing Process on the Profile of Biogenic Amines in Craft Beers

**DOI:** 10.3390/s23010343

**Published:** 2022-12-29

**Authors:** Renato L. Gil, Célia M. P. G. Amorim, Henrique G. Amorim, Maria da Conceição B. S. M. Montenegro, Alberto N. Araújo

**Affiliations:** 1 LAQV-REQUIMTE, Department of Chemical Sciences, Faculty of Pharmacy, University of Porto, R. Jorge Viterbo Ferreira 228, 4050-313 Porto, Portugal; 2 Mathematics Department, Faculty of Sciences, University of Porto, R. Campo Alegre s/n, 4169-007 Porto, Portugal

**Keywords:** food safety, craft beers, biogenic amines, liquid chromatography, potentiometry, ion-selective electrodes

## Abstract

The evaluation of the biogenic amines (BAs) profile of different types of craft beers is herein presented. A previously developed and validated analytical method based on ion-pair chromatography coupled with potentiometric detection was used to determine the presence of 10 BAs. Good analytical features were obtained for all amines regarding linearity (R^2^ values from 0.9873 ± 0.0015 to 0.9973 ± 0.0015), intra- and inter-day precision (RSD lower than 6.9% and 9.7% for beer samples, respectively), and accuracy (recovery between 83.2–108.9%). Detection and quantification limits range from 9.3 to 60.5 and from 31.1 to 202.3 µg L^−1^, respectively. The validated method was applied to the analysis of four ale beers and one lager craft beer. Ethylamine, spermidine, spermine, and tyramine were detected in all analyzed samples while methylamine and phenylethylamine were not detected. Overall, pale ale beers had a significantly higher total content of BAs than those found in wheat pale and dark samples. A general least square regression model showed a good correlation between the total content of BAs and the brewing process, especially for Plato degree, mashing, and fermentation temperatures. Knowledge about the type of ingredients and manufacturing processes that contribute to higher concentrations of these compounds is crucial to ensuring consumer safety.

## 1. Introduction

Beer brewing has been practised for thousands of years by many cultures and continues to be a popular and financially significant practice [1]. The brewing process involves the preparation and boiling of the wort, cooling, fermentation, maturation, filtration and/or stabilization, and packing [2,3]. Although the global market is largely dominated by industrial beers, craft beer is gaining market share based on the wide variety of flavors and high-quality perception [4]. While industrial beers are produced in large-scale breweries and follow standard procedures, craft beer relies on local production in microbreweries that develop new styles with no precedent. Craft beer is generally made with brewed and fermented cereals (usually malted barley) and flavored hops, though interesting and non-traditional raw materials have been often added for distinctiveness. Its nutritional value, aroma, and flavor depend greatly on the proteolytic events that the malt and hop undergo during brewing [5].

In general, beer has been considered a safe beverage from a microbiological point of view due to its low pH, oxygen, and nutrient contents as well as the presence of ethanol, hop bitter compounds, and a suitable content of carbon dioxide [6,7]. However, some bacteria can proliferate during processing and storage, with craft beers being more prone to spoilage than those prepared in large-scale breweries [8], probably because they are less likely to be pasteurized or sterile-filtered. The growth of these microorganisms will cause the appearance of organoleptic defects and undesirable compounds, such as biogenic amines (BAs) [9].

BAs are low-molecular-weight nitrogenous compounds with biological activity. Further insight into chemical structure classes them as aliphatic (cadaverine, putrescine, spermine, and spermidine), aromatic (phenylethylamine and tyramine), and heterocyclic (histamine and tryptamine) amines. While some BAs are essential for many physiological functions, others are considered metabolic by-products found in many fermented foods and beverages such as fish, aged meat, cheese, chocolate, vegetables, wine, and beer [10]. Their presence results from the enzymatic decarboxylation of free amino acids, but also the transamination of aldehydes and ketones [11]. In turn, the ingestion of food with high concentrations of BAs may cause a plethora of damage effects, including migraines, high/low blood pressure, red rash, and gastroenteritis [10,12]. Effects can be even more harmful, such as intracerebral hemorrhage and anaphylactic shock [13], especially if the activity of monoamine oxidase and diamine oxidase enzymes are compromised by ingestion of alcohol, tobacco, or antidepressants [14].

The presence of BAs in beers has become of concern to producers, consumers, and international authorities for reasons of food quality and safety [15,16]. However, no official limits have been set in the European Union (EU) for these compounds. Moreover, the type and concentration of BAs in beer are affected largely by the raw materials and brewing techniques employed in the production process, plus the hygiene conditions maintained [17]. The growing production of craft beers, which are not regulated by standard procedures, imposes the simultaneous analysis of several BAs in these beverages to assess the potential risks to human health.

Several analytical methods for the determination of BAs have been reported by several authors [18,19]. Among them, reversed-phase high-performance liquid chromatography (RP-HPLC) with UV-Vis [20] or fluorimetric [21] detection is the most popular method for accurate quantitative analysis of BAs and is considered a reference methodology by the European Food Safety Authority (EFSA) [15]. However, for these detection processes chemical derivatization of the amine group is required to make the analyte detectable, which involves a laborious process with toxic reagents [22]. Alternatively, direct BAs analysis is feasible by the expensive HPLC-MS/MS tandem technique operated by skilled technicians [23,24]. On the other hand, electroanalytical detectors offer a series of advantages for the determination of BAs, including direct analysis (overcoming laborious pretreatment) with affordable instrumentation and aqueous-based eluents that are non-hazardous for users and the environment [25]. The most used is a conductivity detector coupled to ion chromatography, though a suppressor system is necessary to improve detectability while the lower efficiency of cation exchange columns hinders the complete separation of BAs [26]. To overcome these drawbacks, a potentiometric amine-selective electrode coupled with ion-pair chromatography was recently proposed for the determination of 10 underivatized BAs in alcoholic beverages [27]. The trade-off was the achievement of a fast and eco-friendly procedure with high separation efficiency, in which simple, reliable, and affordable electrodes were easily prepared.

The aim of this work is the determination of aliphatic, aromatic, and heterocyclic BAs in different craft beers commercialized in Portugal using a previously validated method. In addition, a statistical approach was implemented to evaluate the influence of the brewing process on the content of BAs. Overall, this work intended to highlight the competitiveness of potentiometric detection in HPLC for implementation in food quality control laboratories.

## 2. Experimental Section

### 2.1. Chemicals and Reagents

BA standards were obtained, all as hydrochloride salts, from Sigma-Aldrich (Algés, Portugal), which included cadaverine (95%, D22606), ethylamine (98%, ref. 232831), histamine (≥99%, H7250), methylamine (≥98%, ref. M0505), phenylethylamine (≥98%, ref. P6513), putrescine (≥97%, P5780), spermine (≥99%, ref. 85610), spermidine (≥98%, ref. S2501), tryptamine (≥99%, ref. 246557), and tyramine (≥98%, ref. T2879).

The solid-phase extraction (SPE) of BAs was performed using ammonium hydroxide solution (NH_4_OH, ref. 320145) acquired from Sigma-Aldrich (Algés, Portugal) and methanol (MetOH, ref. 1.06035.2500P) from VWR (Amadora, Portugal). Ultra-pure water with conductivity < 0.055 µS cm^−1^ was used (Heal Force, Shangai, China).

The mobile phase was prepared using butane-1-sulfonic acid sodium salt (SBS, ref. 1183030025) from Sigma-Aldrich (Algés, Portugal), glacial acetic acid (CH_3_COOH, ref. 33209) from Fluka (Porto Salvo, Portugal), and acetonitrile (ACN, ref. 1.00029.2500P), of HPLC gradient grade, from VWR (Amadora, Portugal).

The amine-selective electrodes were prepared using graphite powder (<50 µm, ref. 1.04206), Araldite M (ref. 10951), cucurbit [6] uril hydrate (CB [6], ref. 94544), potassium tetrakis(p-chlorophenyl)borate (TCPB, ref. 60591), o-nitrophenyloctyl ether (o-NPOE, ref. 365130), high molecular weight polyvinyl chloride (PVC, ref. 81392), multi-walled carbon nanotubes (MWCNTs, 110–170 nm × 5–9 μm, ref. 659258), and tetrahydrofuran (THF, ref. 186562) purchased from Sigma-Aldrich (Algés, Portugal). The hardener Ren HY 5162 was (ref. 068620205) from Huntsman (Barcelona, Spain).

### 2.2. Mobile Phase and Standard Solutions

CH_3_COOH solution at 10 mmol L^−1^ was prepared weekly in ultra-pure water. The eluent A was prepared by dissolving the SBS powder in the previous solution to a final concentration of 5.0 mmol L^−1^. The eluent B was prepared by diluting the CH_3_COOH in ultra-pure water to a concentration of 1.0 mmol L^−1^ and then mixing with ACN in the proportion of 90:10 (*v*/*v*), respectively. Mixed cellulose ester membranes, hydrophilic, 0.22 µm (ref. GSWP04700) from Millipore (Algés, Portugal) were used to filter the eluents. Stock solutions of BAs were prepared in the CH_3_COOH solution for a concentration of 10.0 mmol L^−1^ and stored in the fridge at 4 °C. Working standard mixtures of BA were prepared by dilution in solvent A before injection.

### 2.3. Craft Beers and Sample Preparation

Five individual craft beers of German origin were purchased from the local market in Portugal (Table 1), including four ale (dark, wheat pale, and two pale) and one lager (red). The bottles were brought to the laboratory and, once there, stored in the fridge at 4 °C and protected from light.

The sample preparation procedure is reported elsewhere [27]. Briefly, 25 mL of craft beer sample were placed into a 50 mL screw cap plastic, degassed by using an ultrasonic bath, and filtered through a 0.2 µm nylon syringe filter (ref. 28145-487, VWR, Amadora, Portugal). Each sample was divided into three aliquots and stored at 4 °C (no more than one week). Further cleanup of each aliquot was performed by SPE using Water Oasis PRIME MCX cartridges (3 cc, 60 mg, ref. 186008918, Waters, Lisboa, Portugal). About 1.0 mL of each sample aliquot was loaded in the cartridge and afterward washed twice with 1.0 mL of ultra-pure water. The BAs were quantitatively recovered after elution in triplicate with 1.0 mL of a MetOH:NH_4_OH solution (95:5, *v*/*v*). Finally, each extract was dried under a nitrogen stream, reconstituted in 1.0 mL of the solvent A of the mobile phase, and further injected three times into the chromatographic system (*n* = 9).

### 2.4. HPLC-Potentiometry Instrumentation and Chromatographic Conditions

The HPLC-potentiometry (HPLC-POT) analysis was performed on a Waters 600 Solvent Delivery Pump (Waters, Milford, MA, USA) equipped with a Rheodyne 7725i six-port external sample injector (IDEX Health & Science, LLC, Middleboro, MA, USA) where a sample loop of 100 µL was connected.

A reversed-phase Luna Omega Polar C18 column (150 × 4.6 mm; 5 µm, Phenomenex) was used as the stationary phase. The elution was performed in gradient mode using solvent A (5.0 mmol L^−1^ SBS in 10.0 mmol L^−1^ CH_3_COOH, pH = 2.5) and solvent B (10% ACN/1.0 mmol L^−1^ CH_3_COOH, *v*/*v*, pH = 2.5). The gradient elution program started with 0% of solvent B for 5 min. Then, solvent B linearly increased to 100% within 5.0 min and remained constant until 17.0 min. Finally, the initial conditions were re-established within 0.5 min and held for 7.0 min to ensure column equilibration. The flow rate was 1.2 mL min^−1^ and the column was kept at a controlled room temperature (±2 °C).

A detailed description of the potentiometric detector can be found elsewhere [27,29]. Briefly, it consisted of a wall-jet flow cell incorporating a handmade amine-selective electrode and a commercial reference electrode (model 6.0727.0 0 0, Metrohm, Switzerland). Both electrodes were connected to a 6-Channel Precision Electrochemistry EMF Interface (LawsonLabs, Inc., Malvern, PA, USA), controlled by graphics software from the same company.

The amine-selective membrane was prepared by dissolving accurately weighed amounts of the ionophore (3.0 mg, CB [6]), ionic additive (0.9 mg, TCPB), polymer (90.0 mg, PVC), plasticizer (200.1 mg, o-NPOE), and ion-to-electron transducer (6.0 mg, MWCNTs) in 3.0 mL of THF. It was applied onto the conductive surface of the miniaturized electrodes by drop-coating 4 × 5 µL with an automatic pipette. Each layer was allowed to dry for 20 min before overnight conditioning (≈12 h) in solvent A of the mobile phase.

### 2.5. Analytical Method Validation

The analytical validation of the proposed method followed the Eurachem [30] and International Conference on Harmonization [31] guidelines.

The linearity was assessed in the range of 1.0–100.0 µmol L^−1^ using seven calibration solutions. The potentiometric signal (E) in mV (i.e., peak height) was firstly plotted as a function of the logarithmic of BAs concentration and fitted by the Nikolsky–Eisenman equation. Afterwards, it was converted in a transformed response tR = 10^E⁄S^ − 1, which was plotted against BA concentration [32]. The coefficients of determination (R^2^) were calculated from the intra-assay calibration curves. The LOD and LOQ were determined by the analysis of standard solutions with decreasing amounts of the analytes until a signal-to-noise ratio of three and ten was reached, respectively. The precision of the potentiometric detector was evaluated by repeatability and intermediate precision using standard solutions of BAs at three concentration levels (1.0, 10.0, and 100.0 µmol L^−1^), analyzed in triplicate. The precision of the whole procedure was similarly evaluated by analyzing replicates of craft beer samples on the same day and three independent days. The obtained results were expressed as relative standard deviation (%RSD). The accuracy was determined by the analysis of spiked beer samples at three fortification levels (1.0, 3.0, and 10.0 µmol L^−1^), which were submitted to the whole analytical procedure. The results were expressed as recovery values accordingly to the following Equation (1):

(1) $$\mathrm{Recovery}\;\left(\%\right)=\frac{{\mathrm{BA}}_{\mathrm{Found}}-{\mathrm{BA}}_{\mathrm{Initial}}}{{\mathrm{BA}}_{\mathrm{Spiked}}}\times 100$$

in which BA_Found_ is the concentration of BA measured in the extracts obtained from the spiked beer samples, BA_Initial_ is the intrinsic concentration of BAs measured in the beer samples, and BA_Spiked_ is the amount of BAs added to the beer samples.

### 2.6. Statistical Analysis

The differences between average concentrations founded on the tested craft beer samples were evaluated by the one-way ANOVA test (at 5% significance level) using the Phyton programming language (version 3.11.0, Python Software Foundation).

A model to evaluate the influence of the brewing process on the content of BAs in craft beers was developed by a general least square regression (GLSR) using R programming language (version 2022.07.2-576, RStudio, PBC). The GLSR was comprised of three independent (fermentation temperature, mashing temperature, and Plato degree) and one dependent (content of total BAs) variable.

## 3. Results and Discussion

### 3.1. Analytical Features of the Method

**Figure 1:**
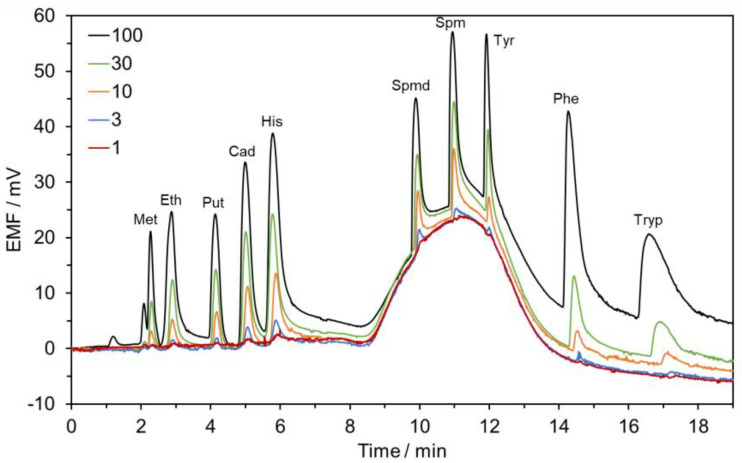
Chromatograms were obtained with the HPLC-POT method for the analysis of standard mixture solutions of BAs. Concentrations are given in µmol L^−1^. Chromatographic conditions: Gradient elution: A—5.0 mmol L^−1^ SBS in 10.0 mmol L^−1^ CH_3_COOH and B—1.0 mmol L^−1^ CH_3_COOH:ACN (*v*/*v*, 90:10). Column: Luna Omega 5 µm Polar C18 150 × 4.6 mm, I.D. (Phenomenex). Flow-rate: 1.2 mL min^−1^. Injection volume: 100 µL.

The following method for the determination of BAs in alcoholic beverages [27] was performed without any modification. Linear regression lines were obtained for putrescine, cadaverine, histamine, spermidine, spermine, and tyramine in the range between 1.0–100.0 µmol L^−1^ whereas for methylamine, ethylamine, phenylethylamine, and tryptamine in the range between 1.0–30.0 µmol L^−1^, with coefficients of determination (R^2^) ranging from 0.9873 to 0.9973, respectively (Table 2). As a representative example, typical chromatograms obtained after the injection of a standard mixture of BAs at ascending concentrations are displayed in Figure 1. LOD and LOQ values were 0.3 and 1.0 µmol L^−1^ for all BAs, which correspond to a range from 9.3 to 60.7 µg L^−1^ and from 31.1 to 202.3 µg L^−1^ attending to the molecular weight of each BA, respectively. The obtained values are in good agreement with those reported in the previous paper [27].

The precision of the potentiometric detector was evaluated by the repeatability (intra-day) and intermediate precision (inter-day) obtained after the triplicate injection of standard mixtures of 10 BAs at different concentrations. The results were expressed as relative standard deviation (%RSD, Table 2). The intra-day precision ranged from 2.7 to 9.5%, 1.9 to 8.5%, and 0.7 to 3.4%, for 1.0, 10.0, and 100.0 µmol L^−1^, respectively. The inter-day precision was evaluated on three independent days and the %RSD values ranged from 4.8 to 9.5%, 3.7 to 8.5%, and 2.0 to 5.6%, respectively.

The accuracy of the whole procedure was evaluated by the analysis of a representative craft beer sample (dark ale) spiked with three concentration levels (1.0, 3.0, and 10.0 µmol L^−1^) and subjected to the whole analytical procedure. The recovery values ranged from 85.3 ± 4.4 to 107.6 ± 4.0%, 86.7 ± 5.7 to 108.9 ± 6.2%, and 83.2 ± 1.9 to 107.7 ± 5.3% for 1.0, 3.0, and 10.0 µmol L^−1^, respectively, with %RSD values lower than 9.2%. These obtained values are in agreement with the European requirements because they fall within the ranges of −20% to +10% for the applied target concentrations [33] and are similar to those reported for industrial beer in the previous work [27]. Methylamine was the amine presenting a lower average percentage recovery (91.2%), while putrescine showed the greatest average recovery (100.7%). Ethylamine and putrescine showed an average recovery slightly higher than 100%, which could be related to some matrix effects since aqueous solutions were used in the construction of calibration curves instead of the sample matrix.

The precision of the whole procedure was evaluated on a similar basis by analyzing one replicate of the craft beer samples on the same day (intra-day precision) and three replicates of the same sample on three independent days (inter-day precision). The %RSD of BA concentration ranged from 0.1 to 7.8% and 1.8 to 9.7% for the intra-day and inter-day precision, respectively (Table 3). Notably, the acceptable %RSD values obtained for each BA indicate the great precision of the method [30].

### 3.2. Biogenic Amines in Craft Beers

Five craft beer samples, namely ale (dark, wheat pale, and pale) and lager (red) types were analyzed. The individual and total levels of BAs are summarized in Table 4, and the amines are ordered by their molecular structure (aliphatic, aromatic, and heterocyclic). The raw material, the alcohol content, and the type/mode of production of each craft beer are shown in Table 1.

**Figure 2:**
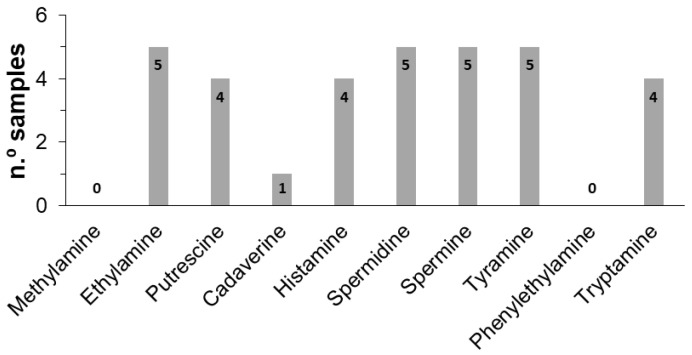
Biogenic amines frequency in the craft beer samples analyzed.

To better understand BAs variability among samples, a statistical study correlating the BAs content with the type of craft beer was made using a one-way Anova test. Comparing the five samples, significant differences were observed (*p* < 0.05) for the total content of BAs (Table 4), which is explained by the different raw materials and processing techniques used in the preparation of craft beers. The higher and lower values were found in beers containing pale ale and wheat pale malts, respectively. Moreover, the total content of BAs in beers produced with pale ale malt was higher when compared to the industrial beers (4.58 mg L^−1^) [27] while in the wheat pale and dark ale types they were slightly lower. As can be observed in Figure 2, ethylamine, spermidine, spermine, and tyramine were found in all analyzed samples, which may be attributed to their presence in hops and barley malt [34]. Putrescine, histamine, and tryptamine were found in more than 50% of the samples while cadaverine was only found in one sample. Methylamine and phenylethylamine were not found in any sample.

Overall, the total content of BAs found in this study is slightly lower than those reported in the literature for craft beer samples commercialized in the central Europe region, with average values of 11.9 mg L^−1^ [35] and 23.7 mg L^−1^ [36]. More data must be collected to support this finding but craft beer is itself a product of modernity in which high technology (including stainless steel, electrical heating, refrigeration, and computer-powered process control) has been enabling a safer food product [16].

Regarding aliphatic amines, the presence of the polyamines spermine and spermidine in all samples might be attributed to the barley malt [34,37] while ethylamine does not seem to be directly related to any raw material [38]. The diamine putrescine was found at lower levels than those reported by other authors (1.59 to 4.05 mg L^−1^ [36]; 2.097 to 12.777 mg L^−1^ [39]; or 3.60 to 8.90 mg L^−1^ [35]), supporting the trend towards higher craft beer quality with modern technology. This amine can be produced by microbial contaminants during fermentation and/or can come from raw materials [40]. On the other hand, cadaverine was only detected in one sample with similar levels to those reported for other craft beers from the European market (0.57 mg L^−1^ [35] and 0.69 mg L^−1^ [36]). It is noteworthy that the low levels of diamines found in this study were indicative of adequate sanitary conditions during the brewing process [15].

Regarding heterocyclic amines, a similar content of histamine was found in four samples, whose levels are in agreement with those reported for craft beers (0.33 mg L^−1^ [36] or 0.017 to 0.339 mg L^−1^ [39]). However, higher values have been reported in the literature from 0.50 to 5.70 mg L^−1^ [35] or 3.8 to 36.6 mg L^−1^ [41]. Despite being the most toxic BA, the amounts found in the analyzed samples do not entail any risk to the health of consumers. Nevertheless, the simultaneous ingestion of beer with amine oxidase inhibitors might increase their toxicity [10]. Tryptamine is present at similar levels to those reported in Chinese beers, ranging from 0.36 to 1.62 mg L^−1^ [42]. This amine is generally absent in craft beers from central European brewers [36,43] or detected with slightly lower levels [35]. The differences in these heterocyclic amines could be related to bacterial contamination during brewing by lactic acid bacteria [43] or other decarboxylative micro-organisms [44].

For aromatic amines, tyramine was found at levels in line with, or lower than, those reported by other authors (0.394 to 5.916 mg L^−1^ [39]; 0.75 to 6.50 mg L^−1^ [35]; 0.17 to 31.60 mg L^−1^ [45]; or 1.2 to 45.1 mg L^−1^ [41]). This amine could be introduced into beers by its intrinsic presence in malt or by its formation during mashing and wort boiling. On the other hand, phenylethylamine was not detected in any analyzed sample, which is in agreement with the low frequency reported for other European beers [35,41].

### 3.3. Influence of the Brewing Process on the Content of Biogenic Amines

To evaluate the influence of the brewing process on the content of BAs, a statistical model was developed using several variables: malt and yeast types, the Plato degree, and mashing and fermentation temperatures. However, the low number of observations obtained from the experimental work only allowed the inclusion of up to three variables for the estimation of the parameters because when minimizing the sum of the squared residuals, more observations than variables are required. In this context, several subsets of the variables were tested and the mashing temperature, fermentation temperature, and Plato degree were those selected for further analysis because a lower Akaike information criterion (AIC) value was obtained.

As the nine observations per beer category were obtained from the same bottle of beer, despite it being divided into three different aliquots (measured three times each), the errors of these observations were surely dependent when fitting the model. For this reason, a general least square regression (GLSR) was used to model not only the correlations amongst the errors but also the heteroscedasticity observed in the data, which is required for more accurate predictions.

The proposed GLSR model included thus two categorical variables (“fermentation temperature” and “mashing temperature”) that were converted to dummy variables (D, between 0 and 1) and one quantitative variable (“Plato degree”) that ranged approximately between 10 and 16. Interactions between the independent variables and the total content of BAs were observed, as demonstrated by Equation (2):

Total BA (mg L^−1^) = 88.31 − 23.45D_1_ − 5.09D_2_ + 19.37D_3_ − 4.21X_1_ (2)

in which D_1_ is the mashing temperature between 66–68 and 76–78 °C, D_2_ is the mashing temperature between 66–69 and 76 °C, D_3_ is the fermentation temperature between 8 and 12 °C, and X_1_ is the Plato degree.

The model presented an excellent *p*-value of <0.0001 for the estimated model coefficients (beta coefficients), which highlight the statistically significant effect of the independent variables on the total content of BAs (Table 5). On the other hand, the good predictive ability of the model is demonstrated by the high correlation (R^2^ > 0.99) and similarity between the observed and predicted values from the model (Table 6).

From Equation (2), the total content of BAs can be easily determined by attending to the brewing conditions. For instance, craft beers with a mashing temperature of 56–68 and 76–78 °C and a fermentation temperature of 18–22 °C (i.e. wheat pale or ale types) have a decrease in the total content of BAs by 4.81 mg L^−1^ by one extra unit of Plato degree, on average. On the other hand, if the fermentation temperature changed to 8–12 °C (especially for lager types) but maintained the mashing temperature, the total content of BAs in the craft beers will be higher by 19.37 mg L^−1^, despite the decrease of 4.81 mg L^−1^ by a unit of Plato degree on average. In contrast, if the fermentation temperature is maintained at 18–22 °C but the mashing temperature changed to 66–69 and 76 °C, the total content of BAs will be lower by 5.09 mg L^−1^ and 4.81 mg L^−1^ by a unit of Plato degree, on average. Overall, the predictive ability of the proposed model gives crucial information about the content of BAs based on the processing conditions and final beer type.

## 4. Conclusions

The implementation of an HPLC-POT method for routine analysis of BAs in craft beers proved to be simple, effective, and affordable. The major advantage relies on the fast determination of BAs without chemical derivatization and using water-based eluents, under a concept of green chemical analysis.

Overall, the total content of amines in the five craft beers was low, ranging from 3.83 to 9.60 mg L^−1^ for wheat pale ale and pale ale types, respectively. Ethylamine, spermidine, spermine, and tyramine were detected in all analyzed samples, followed by putrescine, histamine, and tryptamine, which were detected in more than 50% of the samples. Histamine and tyramine, considered the most toxic and food safety-relevant BAs, were found in low levels (<0.32 and <2.90 mg L^−1^, respectively), which did not entail any risk to the health of consumers.

A GLSR model demonstrated a good correlation between the total content of BAs and the brewing process, especially for Plato degree, mashing, and fermentation temperatures. As microbreweries continue to reinvent the landscape of beer by using different ingredients and techniques, the predictive ability of the proposed model is an important landmark to assess the effect of new recipes on the profile of biogenic amines.

## Figures and Tables

**Table 1:** Information on the craft beer samples analyzed in this work [28].

Sample Name	Beer Type	%Plato	Colour	Malt	Hop	Yeast	Mashing Temp. (°C)	Fermentation Temp. (°C)	Maturation Time (Weeks)
Dark	Ale	16.5	Black	Roasted barley Chocolate Coffee Carafa I and III	-	Ale (*S. cerevisiae*)	66–69 76	18–22	<2
Wheat Pale	Ale	12.7	Red gold	Wheat Pilsner Munich	Citra Hallertauer Blanc	Ale (*S. cerevisiae*)	66–68 76–78	18–22	<2
Pale 1	Ale	16.8	Amber	Pale Ale Munique Vienna Roasted barley	Mosaic Yellow sub	Ale (*S. cerevisiae*)	65 76	18–22	<2
Pale 2	Ale	11.5	Golden yellow	Pale Ale Vienna	-	Ale (*S. cerevisiae*)	66–68 76–78	18–22	<2
Red	Lager	16.5	Red	Red rye crystal Pale Ale Munique Vienna	-	Lager (*S. pastorianus*)	66–68 76–78	8–12	>2

**Table 2:** Linearity, recovery range of BAs for three concentration levels (%), relative standard deviation (%RSD), the limit of detection (LOD), and limit of quantification (LOQ).

BA	Linearity				Precision		Accuracy	LOD	LOQ
	Range (µmol L^−1^)	Slope (Mean ± SD)	Intercept (Mean ± SD)	R² (Mean ± SD)	Intra-Day (%RSD)	Inter-Day (%RSD)	Recovery %	(µg L^−1^)	(µg L^−1^)
Methylamine	1–30	90.5 ± 4.9	−0.023 ± 0.017	0.9910 ± 0.0040	0.7–8.0	2.0–9.0	83.2–100.2	9.3	31.1
Ethylamine	1–30	95.3 ± 2.1	−0.074 ± 0.037	0.9905 ± 0.0037	1.0–8.5	3.3–8.6	94.3–106.7	13.5	45.1
Putrescine	1–100	273.0 ± 5.8	−0.841 ± 0.264	0.9951 ± 0.0020	1.1–9.3	5.6–8.5	90.4–109.6	26.5	88.2
Cadaverine	1–100	378.2 ± 9.5	−0.957 ± 0.272	0.9973 ± 0.0015	2.5–4.1	4.4–4.8	81.1–107.7	30.7	102.2
Histamine	1–100	326.5 ± 7.7	−0.983 ± 0.253	0.9938 ± 0.0019	1.7–9.5	4.6–6.2	81.6–102.7	33.3	111.1
Spermidine	1–100	606.1 ± 4.2	−1.066 ± 0.408	0.9964 ± 0.0055	1.9–9.5	2.9–5.0	92.1–110.5	46.6	145.3
Spermine	1–100	429.2 ± 3.4	−0.920 ± 0.921	0.9945 ± 0.0033	2.5–3.3	5.5–9.5	80.2–108.6	60.5	202.3
Tyramine	1–100	134.0 ± 1.9	−0.634 ± 0.170	0.9878 ± 0.0061	3.4–6.9	2.4–7.0	81.1–108.2	41.2	137.2
Phenyl-ethylamine	1–30	33.1 ± 2.3	−0.047 ± 0.034	0.9873 ± 0.0015	1.6–8.3	3.6–5.7	86.1–106.6	36.4	121.2
Tryptamine	1–30	21.9 ± 1.5	−0.025 ± 0.028	0.9935 ± 0.0056	2.5–6.7	2.8–8.9	80.9–104.4	48.1	160.2

SD: Standard deviation (*n* = 3).

**Table 3:** Intra-day and inter-day precision of the proposed method is expressed in relative standard deviation (%RSD) by the analysis of craft beer samples (*n* = 3).

BA	Intra-Day (%RSD)					Inter-Day (%RSD)				
	Dark	Wheat Pale	Pale 1	Pale 2	Red Lager	Dark	Wheat Pale	Pale 1	Pale 2	Red Lager
Methylamine	N.D.	N.D.	N.D.	N.D.	N.D.	N.D.	N.D.	N.D.	N.D.	N.D.
Ethylamine	0.4	3.0	3.3	7.8	3.0	3.8	8.4	3.8	5.9	9.5
Putrescine	0.3	N.D.	3.2	4.4	0.8	8.8	N.D.	7.2	3.2	8.8
Cadaverine	N.D.	N.D.	N.D.	0.8	N.D.	N.D.	N.D.	N.D.	9.6	N.D.
Histamine	0.4	0.7	0.7	N.D.	1.2	9.7	9.3	7.5	N.D.	1.8
Spermidine	1.9	6.4	3.7	7.2	0.9	7.6	5.8	6.4	6.8	3.9
Spermine	0.9	3.7	6.9	0.8	0.5	9.3	4.9	6.4	9.0	2.1
Tyramine	2.3	2.9	3.7	4.3	2.5	5.3	2.9	2.2	9.1	3.0
Phenyl-ethylamine	N.D.	N.D.	N.D.	N.D.	N.D.	N.D.	N.D.	N.D.	N.D.	N.D.
Tryptamine	2.8	N.D.	0.1	1.0	0.4	4.7	N.D.	8.9	8.8	8.6

N.D. Not detected.

**Table 4:** Biogenic amines content (mg L^−1^) in craft beers (average ± SD, *n* = 9).

Beer Type	Aliphatic						Aromatic		Heterocyclic		Total
	Met	Eth	Put	Cad	Spmd	Spm	Tyr	Phe	His	Tryp	
Ale											
Dark	N.D.	0.32 ± 0.01	0.37 ± 0.03	N.D.	0.18 ± 0.01	0.37 ± 0.03	1.60 ± 0.08	N.D.	0.26 ± 0.02	0.83 ± 0.04	3.93 ± 0.13
Wheat Pale	N.D.	0.35 ± 0.03	N.D.	N.D.	0.75 ± 0.04	0.25 ± 0.01	2.22 ± 0.07	N.D.	0.25 ± 0.02	N.D.	3.83 ± 0.05
Pale1	N.D.	0.49 ± 0.02	0.40 ± 0.03	N.D.	2.13 ± 0.14	0.39 ± 0.02	2.90 ± 0.06	N.D.	0.32 ± 0.02	0.95 ± 0.08	7.65 ± 0.18
Pale2	N.D.	0.26 ± 0.02	0.51 ± 0.02	0.46 ± 0.06	4.79 ± 0.32	0.61 ± 0.05	1.85 ± 0.17	N.D.	N.D.	1.13 ± 0.10	9.60 ± 0.21
Lager											
Red	N.D.	0.28 ± 0.03	0.37 ± 0.03	N.D.	0.31 ± 0.01	0.56 ± 0.01	1.98 ± 0.06	N.D.	0.32 ± 0.01	1.12 ± 0.10	4.94 ± 0.13
*p*-value	-	<0.001	-	-	<0.001	<0.001	<0.001	-	-	-	<0.001

*p*-values from the one-way ANOVA test; Cad: Cadaverine; Eth: Ethylamine; His: Histamine; Met: Methylamine; N.D.: Not detected; Phe: Phenylethylamine; Put: Putrescine; Spm: Spermine; Spmd: Spermidine; Tryp: Tryptamine; Tyr: Tyramine.

**Table 5:** Statistical significance of estimated model coefficients.

Term	Coefficient	Std. Error	t-Value	*p*-Value
Intercept	88.3114	2.8168	31.3517	8.93 × 10^−30^
D_1_	−23.4458	0.7062	−33.2004	9.80 × 10^−31^
D_2_	−5.0931	0.1203	−42.3201	7.77 × 10^−35^
D_3_	19.3711	0.6530	29.6629	7.48 × 10^−29^
X_1_	−4.8056	0.1676	−28.6771	2.72 × 10^−28^

**Table 6:** Total content of BAs (mg L^−1^) observed for craft beers and predicted values from the proposed GLSR model.

Beer Type	Observed (Max/Min)	Predicted	Residuals (Max/Min)
Ale			
Dark	4.103/3.766	3.925	0.177/−0.159
Wheat Pale	3.906/3.758	3.834	0.072/−0.076
Pale1	7.819/7.353	7.577	0.242/−0.224
Pale2	9.742/9.073	9.601	0.141/−0.528
Lager			
Red	5.704/4.722	4.944	0.130/−0.222

## Data Availability

Not applicable.
